# The role of ferroptosis in colorectal cancer and its potential synergy with immunotherapy

**DOI:** 10.3389/fimmu.2024.1526749

**Published:** 2025-01-09

**Authors:** Wenhua Xia, Yuanhao Lv, Yan Zou, Zhanting Kang, Zhaoyi Li, Jiaqi Tian, Hongyan Zhou, Wei Su, Jiateng Zhong

**Affiliations:** ^1^ Department of Pathology, School of Basic Medical Sciences, Xinxiang Medical University, Xinxiang, China; ^2^ Department of Pathology, The First Affiliated Hospital of Xinxiang Medical University, Xinxiang, China; ^3^ Xinxiang Key Laboratory of Precision Diagnosis and Treatment for Colorectal Cancer, Xinxiang First People’s Hospital, Xinxiang, China; ^4^ Xinxiang Engineering Technology Research Center of Digestive Tumor Molecular Diagnosis, The First Affiliated Hospital of Xinxiang Medical University, Xinxiang, China

**Keywords:** ferroptosis, colorectal cancer, immunotherapy, immune checkpoint inhibitors (ICBs), tumor microenvironment

## Abstract

Colorectal cancer (CRC) is one of the most prevalent and deadly malignancies worldwide. Recently, ferroptosis, a novel form of regulated cell death characterized by iron dependency and lipid peroxidation, has garnered significant attention from researchers. The mechanisms underlying ferroptosis, including intracellular iron levels, lipid peroxidation, and antioxidant system regulation, offer new insights into cancer treatment strategies. This study aims to explore the emerging role of ferroptosis in the context of immunotherapy for CRC, highlighting its potential mechanisms and clinical applications. We employed a comprehensive review of current literature to elucidate the biological mechanisms of ferroptosis, its relationship with CRC, and the interplay between ferroptosis and immunotherapy. Ferroptosis reshapes the tumor microenvironment (TME) by regulating intracellular iron levels, lipid metabolism, and antioxidant systems, significantly enhancing the efficacy of immune checkpoint inhibitors (ICIs). Meanwhile, traditional Chinese medicine therapies promote antitumor immunity by modulating the TME and inducing ferroptosis. Additionally, advances in nanotechnology have facilitated precise therapy by enabling targeted delivery of ferroptosis inducers or immunomodulators, transforming “cold” tumors into “hot” tumors and further boosting ICI efficacy. This study comprehensively reviews the latest developments in ferroptosis, immunotherapy, traditional Chinese medicine, and nanotechnology in CRC, highlighting the importance of ferroptosis-related biomarkers and novel inducers for personalized treatment. In summary, ferroptosis offers a promising strategy to overcome CRC therapy resistance and enhance immunotherapy efficacy, warranting further investigation and translational application.

## Introduction

Globally, colorectal cancer is the third most common malignant tumor in terms of incidence, with a mortality rate of 9.3% of the total cancer prevalence worldwide (1). Although early screening has been shown to be effective in reducing morbidity and mortality in the general population, approximately 25% to 50% of patients with early-stage colorectal cancer are still at risk for tumor metastasis, and approximately 25% of patients are in advanced stages at the time of diagnosis (2, 3). These factors seriously affect treatment outcome and survival prognosis (4–6). In recent years, the age of onset of colorectal cancer has shown a tendency to be younger, and studies have shown that the incidence of colorectal cancer in young and middle-aged people under the age of 50 has continued to rise since the mid-1980s (7). The phenomenon may be closely related to factors such as the widespread use of antibiotics,intestinal flora dysbiosis increased obesity rates, and other factors (7–9). Currently, significant progress has been made in the treatment of colorectal cancer, including surgical resection, radiation therapy, targeted therapy, chemotherapy and immunotherapy. For early-stage colorectal cancer patients, radical surgery combined with adjuvant radiotherapy and chemotherapy can usually achieve a better prognosis and a higher five-year survival rate (10). However, for patients with metastatic, malignant or invasive colorectal cancer, the effectiveness of existing treatments remains limited and the prognosis is poor (11). Therefore, colorectal cancer has become a major public health challenge that needs to be addressed globally. Improving early detection rates and optimizing treatment strategies, especially in advanced metastatic cases, remains a major challenge in the current treatment of colorectal cancer. There is an urgent need to develop new and more effective treatments to improve the survival prognosis of patients.

Cell death plays a pivotal role in development and physiological homeostasis, being essential in embryogenesis and mechanisms like immune surveillance and tissue repair in adults. According to the Nomenclature Committee on Cell Death (NCCD), cell death is classified into accidental cell death (ACD) and regulated cell death (RCD) (12). ACD arises from unpredictable damaging stimuli exceeding repair capacity, while RCD is a gene-regulated process mediated by signal amplification complexes critical for development and homeostasis. Programmed cell death (PCD), a subtype of RCD, removes aberrant, aging, or damaged cells under physiological conditions, maintaining immune and tissue balance (13). Dysregulation of cell death mechanisms can lead to pathological conditions, such as excessive neuronal apoptosis in neurodegenerative diseases or failure to eliminate mutated cells in tumorigenesis, highlighting its dual role in disease progression (14–16).

In recent years, the classification of cell death has been further refined, leading to the emergence of new types of cell death such as autophagic death, pyroptosis, and cupric death, which have unique physiological and pathological characteristics. Among these novel modes of cell death, ferroptosis, a non-apoptotic programmed cell death mode dependent on iron, has gradually attracted widespread attention. Ferroptosis, first proposed in 2012, is a novel programmed cell death mechanism that is mainly triggered by iron-dependent lipid peroxidation and the accumulation of reactive oxygen species (ROS) (17). This mechanism differs from conventional apoptosis in that ferroptosis is characterized by distinctive cellular morphological changes, such as a marked reduction in the size of mitochondria, a decrease in the number of mitochondrial cristae, and an increase in the density of membranes (17, 18).

Research on ferroptosis has revealed its potential role in various pathological states, particularly in diseases such as intestinal disorders, neurodegenerative diseases, and cardiovascular diseases, where ferroptosis is believed to be a key factor in pathological progression (15, 19–22). Therefore, regulating iron metabolic pathways, especially by inducing ferroptosis, has become a new direction for treating tumors. By modulating the ferroptosis pathway, researchers hope to develop innovative therapeutic approaches for targeting tumor cells, especially when conventional therapies fail or develop resistance. As an emerging mechanism of cell death, ferroptosis could serve as an alternative therapeutic strategy for tumor cells that are insensitive to conventional chemotherapy, including how to improve the selectivity of ferroptosis inducers and avoid side effects on normal tissues (15, 19–23).

Immunotherapy has become an important direction in the treatment of colorectal cancer in recent years. Immune checkpoint inhibitors (ICIs), such as PD-1/PD-L1 and CTLA-4, restore and enhance the body’s immune surveillance and attack ability against tumors by relieving the inhibition of tumor cells on the immune system (24, 25). However, the efficacy of existing immunotherapy is still limited for patients with microsatellite stable (MSS) or other immunotherapy-resistant colorectal cancer, so there is an urgent need to find new combination treatment strategies to improve the efficacy.

Some studies have shown a strong link between ferroptosis and immunotherapy. Ferroptosis-related oxidative stress and cell death mechanisms may enhance the efficacy of immunotherapy by influencing immune escape mechanisms in the tumour microenvironment (17, 18, 26). In particular, immune checkpoint inhibitors have shown better efficacy in microsatellite unstable (MSI-H) colorectal cancer, especially in microsatellite stable (MSS) colorectal cancer, where efficacy is still unsatisfactory. However, in patients with microsatellite-stable (MSS) or other immunotherapy-resistant colorectal cancers, the efficacy of existing immunotherapies is still limited (27, 28). Therefore, researchers are actively exploring new therapeutic strategies, such as combination chemotherapy, radiotherapy, targeted therapies, and novel ferroptosis-based therapies, with the aim of improving the response rate and efficacy of immunotherapy, and bringing more effective treatment options to colorectal cancer patients.

## Novel mechanism to regulate cell death: ferroptosis

The goal of most cancer treatment strategies is to selectively eliminate cancer cells without damaging non-malignant cells. Different sub-programs of programmed cell death (RCD) have different effects on tumor progression and treatment response (29). In contrast to accidental cell death, RCD is controlled by specific signaling pathways that can be intervened by pharmacological or genetic means. One such ferroptosis is a type of iron-dependent RCD.

**Figure 1:**
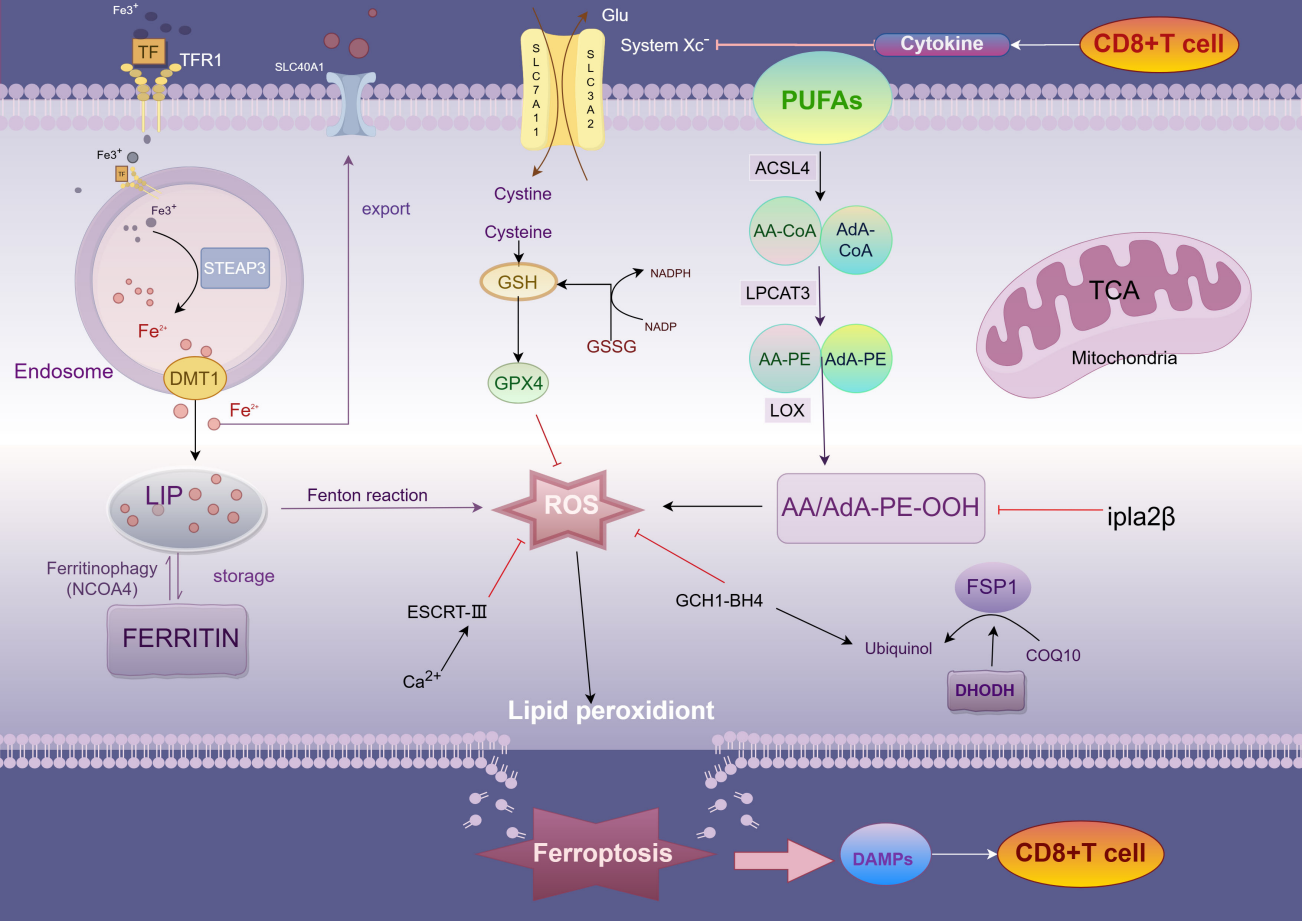
Mechanism of ferroptosis. TFR1, transferrin receptor 1; DMT1, divalent metal transporter 1; peptidase-4 STEAP3, six-transmembrane epithelial antigen of prostate; LIP, labile iron pool; PUFA, polyunsaturated fatty acids; ACSL4, acyl-CoA synthetase long-chain family member 4; AA, arachidonic acid; AdA, adrenic acid; LPCAT3, lysophosphatidylcholine acyltransferase 3; LOX, lipoxygenases; System Xc−, cystine-glutamate exchange system; FSP1, ferroptosis suppressor protein 1.

In 2012 Dixon et al. named the discovery of a unique form of cell death by the first induction of the small molecule compound erastin as ferroptosis (17). Morphologically, ferroptosis cells show typical necrotic features, including shrinkage of mitochondria, reduction of cristae, increase in membrane density, and rupture of the outer membrane, but are not accompanied by the typical hallmarks of apoptosis (12). Further studies revealed that ferroptosis is mediated by mitochondrial voltage-dependent anion channels (VDACs); specifically, erastin promotes mitochondrial uptake of ferric ions, reactive oxygen species (ROS) generation, elevated mitochondrial potential, and oxidative stress, thereby triggering ferroptosis. Studies over the years have demonstrated that mitochondria play a key role in ferroptosis, affecting the onset of ferroptosis through the modulation of mitochondrial lipid metabolism, energy metabolism, and iron metabolism, among others (18). In addition to the mitochondrion, other organelles such as the endoplasmic reticulum, the Golgi, and lysosomes are also participate in the regulation of ferroptosis, which they promote through oxidative stress, lipid peroxidation, and dysfunction, respectively (21, 23). In the presence of the antioxidant glutathione (GSH), glutathione peroxidase 4 (GPX4) exhibits a unique ability to inhibit lipid peroxidation and protect the cells from ferroptosis. This finding reveals a complex interaction between iron, cysteine, and lipid metabolism as an important regulatory mechanism for ferroptosis and a series of related regulators have been identified. Because cancer cells require large amounts of iron to meet their metabolic demands and promote growth, they are more susceptible to ferroptosis (30, 31). Since the discovery of ferroptosis, a large number of studies have targeted ferroptosis for its anticancer potential, and these effects may be mediated through immune-mediated mechanisms (**Figure 1**).

## Mechanisms and signaling pathways of ferroptosis

### Mechanisms of lipid peroxidation

Peroxidation of lipid components is the central driving mechanism of ferroptosis. In this process, accumulation of lipid bilayer peroxides (PLOOHs) irreversibly triggers ferroptosis when it exceeds the antioxidant capacity of the cell (32). Acyl coenzyme A synthetase long-chain family member 4 (ACSL4) and lysophosphatidylcholine acyltransferase 3 (LPCAT3) play key roles in this process and are responsible for the synthesis, activation, and incorporation of polyunsaturated fatty acid phospholipids (PUFA-PLs), whereas arachidonic acid (AA) and adrenoic acid (AdA), which are the most susceptible lipids to peroxidation, are present in PUFA. ACSL4 forms AA -CoA or AdA-CoA by catalyzing the attachment of free PUFA to coenzyme A (CoA), which is subsequently esterified to membrane phosphatidylethanolamine (PE) by LPCAT3 and integrated into the cell membrane structure (26). As the multiple double-bond structure of PUFA is susceptible to attack by reactive oxygen species (ROS) generated by the Fenton reaction, lipid peroxides (LPO) are formed. With the accumulation of LPO, the cell membrane structure is impaired, ultimately inducing ferroptosis (23). In addition, deletion of ACSL4 or, more recently, P53 has been shown to activate the expression of calcium-independent phosphatase A2β (Ipla2β).

Ipla2β is an enzyme capable of hydrolyzing lipid peroxidation, which hydrolyzes oxidized PUFA-PLs from the phospholipid molecules of the cell membrane and reduces the accumulation of lipid peroxidation, thus inhibiting ferroptosis to a certain extent (33). Not only that, as lipid peroxidation continued to accumulate, protein kinase CβII (PKCβII) was activated and interacted with the Thr328 site of phosphorylated ACSL4, increasing the production of PUFA-PLs and further inducing ferroptosis (33, 34). Notably, accumulation of lipid peroxides sustains activation of PKCβII, creating a positive feedback loop that in turn enhances the sensitivity of tumor cells to immune-mediated killing.

## Antioxidant defenses

The Xc-system consists of two subunits, SLC7A11 and SLC3A2. Under normal homeostatic conditions of the internal environment, lipid peroxides (PLOOH) in cells are reduced to nontoxic lipid alcohols through the glutathione (GSH)-glutathione peroxidase 4 (GPX4) antioxidant system (i.e., the Xc-system) (26, 34). This reduction process effectively prevents further accumulation of lipid peroxides and avoids overproduction of reactive oxygen species (ROS), thereby inhibiting oxidative stress and ferroptosis.GPX4, an important regulator of ferroptosis, maintains the integrity and function of cell membranes through its own antioxidant action by reducing peroxisomal phospholipids to hydroxyphospholipids form (31, 35, 36). However, when intracellular GSH is depleted or GPX4 is inactivated due to certain factors (e.g., ubiquitination, etc.), lipid peroxides are no longer efficiently scavenged and begin to accumulate in large amounts in the cell membrane (30). Since the structure of PUFA-PL contains multiple double bonds, it is susceptible to ROS attack, leading to the peroxidation chain reaction. This autocatalytic process exacerbates lipid oxidation, which ultimately destroys the structure and function of the cell membrane, resulting in an increase in membrane permeability, loss of membrane potential, and a complete breakdown of intracellular homeostasis. When lipid peroxidation reaches a certain critical value, the whole lipid peroxidation system enters an uncontrolled state, and the cell is exposed to intense oxidative stress pressure, which ultimately triggers ferroptosis. The oxidative damage in this process not only severely destabilizes the cell membrane, but also promotes irreversible cell death through the activation of various downstream signaling pathways, such as apoptosis-related signals, inflammatory response pathways, and autophagy pathways. Erastin, an inhibitor of ferroptosis, induces ferroptosis by inhibiting the function of the Xc- system (17, 23). Not only that, cisplatin also induced ferroptosis in colorectal cancer A549 and HCT116 cells, and the combination of cisplatin and Erastin increased the antitumor activity even more (37).

High expression of GPX4 in different tumor cells has different survival outcomes. For example, in pancreatic cancer cells, high expression of GPX4 was positively correlated with survival, whereas high expression in colorectal cancer cells had a poor survival outcome (36, 38, 39). Thus, GSH depletion as well as functional imbalance of GPX4 may be a potential strategy to improve cancer treatment outcomes. In addition to the GSH-GPX4 system, there are other antioxidant pathways in cells that counteract ferroptosis. For example, tetrahydrobiopterin (BH4), an endogenous antioxidant, inhibits ferroptosis by selectively preventing the depletion of phospholipids containing two PUFA tails through the expression of its GTP cyclase hydratase 1 (GCH1), and GCH1 is also associated with an increase in CoQ10 synthesis through the BH4-mediated phenylalanine to tyrosine conversion (40). Whereas Ferroptosis suppressor protein 1 (FSP1, also known as apoptosis-inducing factor mitochondrial 2 AIFM2) acts as an oxidoreductase in the cell membrane, reducing ubiquinone 10 to ubiquinol (CoQ10H2) with NADPH as the electron donor, ubiquinol can act as a lipid-soluble RTA, thus inhibiting lipid peroxidation and preventing ferroptosis (41, 42). In CRC, FSP1 is inhibited by. Not only that, the sorting complex (ESCRT-III) in endosomes, as well as by promoting cell membrane repair, was also able to partially alleviate the membrane damage triggered by ferroptosis. And in colorectal cancer cells, especially in tumors, these antioxidant systems act synergistically through different mechanisms to help cancer cells resist excessive oxidative stress and slow down ferroptosis (23). Thus, dysregulation of the antioxidant system is closely related to the induction of ferroptosis, especially in tumor therapy, and modulation of these pathways promises to be a new therapeutic strategy.

## Iron metabolism pathway

Iron metabolism is closely related to the process of ferroptosis, which is characterized by iron-dependent lipid peroxidation. Under normal physiological conditions, iron is transported in the blood as Fe³^+^ bound to transferrin. Transferrin receptor 1 (TFR1) mediates the uptake of Fe³^+^, which is followed by the reduction of ferrous iron in acidic endosomes in response to prostate six transmembrane epithelial antigen 3 (STEAP3), which in turn passes through the divalent metal transporter protein 1 (DMT1) or enters the cytoplasmic pool of unstable iron that stores reactive iron (LIP) (43, 44). Intracellular iron is mainly stored in ferritin, and nuclear receptor coactivator 4 (NCOA4) binds directly to ferritin heavy chain (FTH1), mediating ferritin degradation through the autophagy pathway (also known as Ferritinophagy) and releasing iron into the LIP (45, 46). Inhibition of NCOA4 reduces iron content in LIP and prevents ferroptosis, whereas enhancement of ferritin autophagy increases iron content in LIP and promotes ferroptosis in CRC cells. The expression level of NCOA4 is positively correlated with sensitivity to ferroptosis inducers (45). Specific small molecule inhibitor 9a significantly improves the clinical prognosis of acute myeloid leukemia (AML) by targeting the interaction between NCOA4 and FTH1, leading to depletion of iron content in LIP, thereby inhibiting ferritin autophagy, blocking lipid peroxidation and ferroptosis, and exerting an inhibitory effect on the self-renewal and viability of leukemic stem cells (47, 48). Not only that, compound 9a demonstrated a significant protective effect against ischemia-reperfusion injury (47). Once intracellular free iron accumulates, excess Fe²^+^ reacts with hydrogen peroxide (H_2_O_2_) via the Fenton reaction to produce ROS (mainly highly reactive hydroxyl radicals), which triggers lipid peroxidation and membrane damage, inducing ferroptosis. The regulation of hepcidin (iron regulator) affects iron release and reutilization. FINO2 and other novel ferroptosis-inducing agents that trigger the generation of lipid peroxides by oxidizing Fe²^+^ to Fe³^+^ represent a fourth class of ferroptosis-inducing compounds.

## Association between colorectal cancer and ferroptosis

Ferroptosis, an iron-dependent form of programmed cell death, has garnered significant attention in recent years. Its regulation plays a critical role in tumorigenesis. Dysregulation of ferroptosis during tumor progression leads to increased oxidative stress within the tumor microenvironment, promoting invasion, metastasis, and immune evasion (49–51). In colorectal cancer, ferroptosis is a key factor influencing tumor progression and clinical prognosis (52–55). It regulates cell growth, migration, invasion, and chemosensitivity, thereby shaping clinical outcomes. For instance, the expression of ferroptosis-related genes such as TFAP2C, ACACA, and NOS2 correlates significantly with patient survival, as demonstrated by receiver operating characteristic (ROC) and Kaplan-Meier (K-M) analyses, even surpassing traditional TNM staging in prognostic assessment (56–58). Further studies indicate that ferroptosis sensitivity is closely linked to metabolic reprogramming in tumor cells. Recent findings reveal a connection between mTORC1 and ferroptosis, with mTORC1 acting as a critical regulator (59, 60). Notably, the combination of aspirin and RSL3 effectively induces ferroptosis in PIK3CA-mutant CRC cells by inhibiting the mTOR/SREBP-1/SCD1 signaling pathway (61). Thus, ferroptosis emerges as a multifaceted mechanism in CRC pathogenesis and progression. Investigating its molecular mechanisms, particularly identifying ferroptosis-related biomarkers, could pave the way for novel approaches in early diagnosis and personalized treatment strategies.

## The role and application of ferroptosis in CRC immunotherapy

### Mechanisms of ferroptosis in CRC immunotherapy

The resistance to tumor immunotherapy is primarily associated with the immunosuppressive characteristics of the tumor microenvironment (TME). In CRC, the TME is enriched with immunosuppressive cells such as tumor-associated macrophages (TAMs) and regulatory T cells (Tregs), which secrete inhibitory cytokines and express immune checkpoint molecules like PD-L1, suppressing T-cell activity and evading immune surveillance (62, 63). Ferroptosis significantly influences the dynamic equilibrium of the TME by regulating the function of immune cells (23). TAMs, dendritic cells (DCs), and Tregs in CRC are particularly sensitive to ferroptosis, highlighting its pivotal role in both tumor metabolism and the modulation of immune responses.

## CD8^+^ T cells

As core effector cells in antitumor immunity, CD8+ T cells enhance tumor cell lysis by secreting interferon-γ (IFN-γ), which inhibits the expression of SLC7A11 and SLC3A2—subunits of the system Xc^–^ in tumor cells. This suppression reduces cystine uptake, depletes glutathione levels, and induces lipid peroxidation, ultimately triggering ferroptosis (64–66). The resulting tumor cell lysis releases damage-associated molecular patterns (DAMPs) such as ATP, HMGB1, and CRT, which activate innate immunity and amplify antitumor responses (67, 68). Additionally, studies have identified apolipoprotein L3 (APOL3) as a key player in CRC, enhancing CD8+ T-cell antitumor activity by promoting ubiquitin-mediated degradation of lactate dehydrogenase A (LDHA), making it a potential biomarker for ferroptosis-based immunotherapy (69).

Conversely, ferroptosis can contribute to CD8+ T-cell exhaustion. In the TME, lipid uptake and intracellular lipid peroxidation accumulate in infiltrating CD8+ T cells, leading to ferroptosis-mediated dysfunction (70, 71). Oxidized low-density lipoproteins (OxLDL) in the TME, taken up by CD36-expressing CD8+ T cells, increase lipid peroxidation and activate stress response protein p38, triggering ferroptosis. This process reduces the expression of IFN-γ and TNF-α, impairing CD8+ T-cell function and weakening antitumor efficacy. Additionally, CD36 expression correlates positively with immune checkpoint proteins PD-1 and TIM-3. Overexpression of antioxidant enzyme GPX4 can inhibit OxLDL-induced lipid peroxidation, preserving T-cell function and restoring antitumor immunity (72, 73). Furthermore, APOL3 overexpression enhances the synergistic effects of ferroptosis inducers like RSL3 and PD-1 inhibitors in CRC (69). Therefore, ferroptosis in the CRC microenvironment exerts multifaceted regulatory effects on CD8+ T cells, facilitating tumor immune clearance through diverse mechanisms.

## Treg cells

Regulatory T cells (Tregs) play a crucial role in maintaining immune tolerance and facilitating tumor immune evasion. The expression of GPX4 in Tregs is essential for resisting oxidative stress within the TME (31). Studies have demonstrated that in MC38 colorectal cancer and B16F10 melanoma models, specific deletion of GPX4 in Tregs leads to significant accumulation of lipid peroxides (LPOs), inducing ferroptosis upon TCR/CD28 co-stimulation and impairing Treg function. GPX4-deficient Tregs are highly susceptible to ferroptosis, which alters immune homeostasis in the TME by enhancing pro-inflammatory cytokine interleukin-1β (IL-1β) secretion. IL-1β promotes the differentiation and activation of Th17 cells, creating a pro-inflammatory environment. This inflammation further activates dendritic cells (DCs), improving antigen presentation, and enhances the cytotoxic activity of CD8+ T cells. These effector cells are central to antitumor immunity, significantly boosting immune clearance of tumor cells (32). Therefore, GPX4 is vital in regulating oxidative stress resistance and immunosuppressive functions of Tregs, maintaining immune tolerance within the TME.

## TAMs

TAMs are the most abundant innate immune cells in the TME, comprising pro-tumorigenic M2 macrophages and pro-inflammatory, antitumorigenic M1 macrophages (74). While M1 and M2 macrophages exhibit similar LPO levels and expression of key metabolic regulators (e.g., ACSL4, LPCAT3, GPX4), M1 macrophages display greater ferroptosis resistance due to high inducible nitric oxide synthase (iNOS) expression. Conversely, M2 macrophages are more susceptible to ferroptosis. Ferroptosis not only induces direct tumor cell death through lipid peroxidation but also reshapes macrophage phenotypes within the TME. This process drives the transition of M2 macrophages toward the antitumor M1 phenotype, enhancing immune response efficacy (75). For instance, the natural compound dictamnine inhibits ERK phosphorylation and suppresses M2 TAMs, thereby inducing ferroptosis in CRC cells (76).

**Figure 2:**
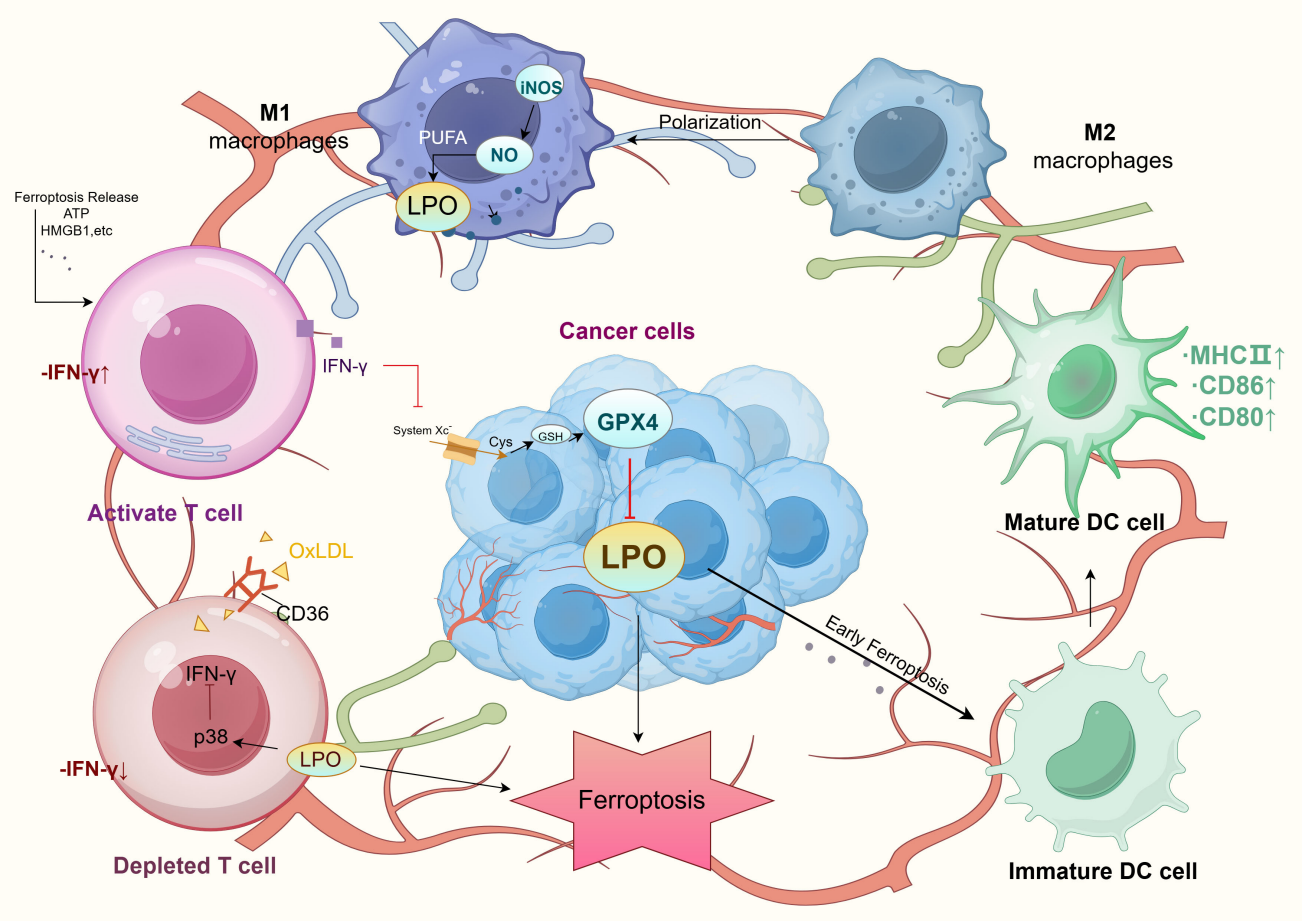
Tumor microenvironment and ferroptosis mechanism. LPO, Lipid Peroxidation; PUFA, Polyunsaturated Fatty Acids; iNOS, Inducible Nitric Oxide Synthase; NO, Nitric Oxide; GPX4, Glutathione Peroxidase 4; GSH, Glutathione; Cys, Cysteine; System Xc-, Amino acid transport system; OxLDL, Oxidized Low-Density Lipoprotein; MHC II, Major Histocompatibility Complex Class II; DC cell, Dendritic Cell.

Additionally, increased intracellular iron and ROS levels elevate TNF-α expression, promoting macrophage polarization toward the M1 phenotype and further enhancing ferroptosis-mediated cytotoxicity (77). Ferroptosis-induced macrophages secrete pro-inflammatory cytokines such as TNF-α and IL-6, activating other immune cells in the TME and amplifying antitumor immune responses (74). These dual effects of TAMs in ferroptosis regulation highlight novel avenues for tumor immunotherapy (**Figure 2**).

## DCs

Ferroptosis impacts tumor immunity not only by inducing tumor cell death but also by modulating immune cell activity and cytokine secretion, shaping the TME (78). In the early stages of ferroptosis, tumor cells release damage-associated molecular patterns (DAMPs), such as high-mobility group box 1 (HMGB1) and ATP. These molecules significantly enhance dendritic cell (DC) maturation and antigen-presenting capability, activating specific T-cell responses. DAMP release also promotes DC migration to tumor sites, increasing antigen presentation efficiency and reducing immune evasion. Within the TME, DAMPs facilitate antitumor immunity and contribute to the transformation of “cold” tumors into “hot” tumors.

However, in the late stages of ferroptosis, although dying cells are effectively phagocytosed, DC antigen-presentation capacity declines, limiting sustained immune responses. This highlights the dual effects of ferroptosis on the TME, presenting both stimulatory and inhibitory influences. Regulation targeting specific stages of ferroptosis may offer novel strategies to enhance immunotherapy efficacy, especially in colorectal cancer, by optimizing ferroptosis-associated immune modulation to inhibit tumor progression and immune escape (79, 80) (**Figure 2**).

## MDSCs

Myeloid-derived suppressor cells (MDSCs), originating from bone marrow mesenchymal stem cells, are key immunosuppressive cells in the TME (81). They facilitate tumor immune evasion and suppression through metabolic reprogramming and immune regulation (82). Research indicates that N-acylsphingosine amidohydrolase 2 (ASAH2) is upregulated in MDSCs in colorectal cancer, enhancing heme oxygenase-1 (Hmox1) expression via p53 destabilization. This reduces lipid reactive oxygen species (ROS) production and inhibits ferroptosis, maintaining MDSC activity and immunosuppressive function in the TME. The specific ASAH2 inhibitor, NC06, suppresses ceramidase activity, increases lipid ROS, and induces ferroptosis in MDSCs. In tumor-bearing mouse models, NC06 treatment reduced MDSC infiltration and activated tumor-infiltrating cytotoxic T lymphocytes (CTLs), leading to tumor growth inhibition (83). Thus, NC06-mediated MDSC ferroptosis offers a novel therapeutic approach to reduce MDSC infiltration and enhance antitumor immunity within the TME.

## The combination of ferroptosis inducers and immunotherapy

Immunotherapy represents a revolutionary breakthrough in cancer treatment, particularly in disrupting tumor immune evasion mechanisms. The immune system combats cancer by recognizing and eliminating tumor cells, but tumors often evade this through immune checkpoint molecules such as PD-1, PD-L1, and CTLA-4 (84). Immune checkpoint inhibitors (ICIs), as an emerging strategy for colorectal cancer treatment, have shown promising clinical applications in recent years (85, 86). ICIs enhance the anti-tumor immune response by inhibiting the immunosuppressive pathway between tumor cells and TME, and relieving the immune escape of tumor cells from T cells. Combining ferroptosis inducers with ICIs demonstrates synergistic effects by enhancing tumor immunogenicity and reversing immune suppression within the TME. This approach transforms immune-suppressive TME into an inflammatory environment rich in antitumor immune cells, particularly improving the response to “cold” tumors (87, 88). In microsatellite-stable (MSS) colorectal cancer patients, ICIs are less effective due to low mutational burden and reduced neoantigen expression, resulting in limited immunogenicity. Exploring ICIs in combination with other therapies, including ferroptosis inducers, is a critical avenue for improving CRC immunotherapy outcomes.

In recent years, the role of ferroptosis in CRC immunotherapy has garnered significant attention. Ferroptosis inducers such as RSL3 and Erastin have been shown to enhance tumor immunogenicity by directly or indirectly activating ferroptosis pathways (39, 89). Erastin induces ferroptosis by inhibiting the system Xc^−^ and depleting glutathione, while sorafenib, a multi-target tyrosine kinase inhibitor, triggers ferroptosis by suppressing GPX4 activity, synergizing with ICIs like pembrolizumab and nivolumab (90). These combinations not only recruit effector T cells but also convert “cold” tumors to “hot” tumors, improving therapeutic efficacy. For instance, RSL3 enhances PD-1 blockade efficacy by targeting GPX4 to induce lipid peroxidation in CRC cells. Similarly, combining vitamin C (VitC) with cetuximab, an EGFR-targeting antibody, suppresses drug-resistant cell emergence, restricts CRC organoid growth, and delays acquired resistance development in CRC xenografts. Cetuximab impairs glucose metabolism while VitC disrupts iron homeostasis and elevates ROS levels to potentiate ferroptosis (91). Ferroptosis also enhances the sensitivity of CRC to ICIs. For example, Chen c et al. showed that in CRC cells, the expression of CYP1B1 was negatively correlated with that of ACSL4. CYP1B1 activated the PKC signaling pathway by degrading ACSL4, increased the expression of FBXO10, and promoted the ubiquitination and degradation of ACSL4, thereby making tumor cells resistant to ferroptosis. In addition, inhibition of CYP1B1 increased the sensitivity of CRC to anti-PD-1 antibodies and enhanced the response of CRC patients to anti-PD-1 therapy (92). A novel ferroptosis inducer, N6F11, specifically degrades GPX4 via TRIM25-mediated ubiquitin-proteasome pathway activation, triggering ferroptosis in CRC cells. HMGB1 released from ferroptotic cells acts as a DAMP, activating antitumor CD8+ T-cell responses. Additionally, N6F11 suppresses the mTOR/SREBP-1/SCD1 pathway to boost checkpoint blockade therapy (93).

Notably, Traditional Chinese Medicine (TCM) demonstrates significant potential in oncology due to its multi-target, multi-pathway mechanisms, structural stability, and safety profile (94). Active molecules in TCM, such as artemisinin, baicalein, and tanshinone, have been shown to exert antitumor effects by modulating ferroptosis. TCM enhances the immune system and TME immune signaling pathways, showing promise as an adjuvant to ICIs, particularly in CRC.Unlike conventional therapies focusing on direct tumor cell cytotoxicity, TCM leverages TME immune signaling modulation to stimulate immune responses, suppress tumor progression, and improve immunity (95, 96). For instance, ginsenoside Rh3 (GRh3) induces ferroptosis and pyroptosis in CRC cells via the Stat3/p53/NRF2 axis, reducing SLC7A11 expression, depleting intracellular GSH, and increasing ROS and MDA accumulation, thus enhancing immunotherapy efficacy (97). BXD (Banxia Xiexin Decoction) targets the PI3K/AKT/mTOR axis, inducing CRC ferroptosis by increasing intracellular iron and ROS. Similarly, Wei-Tong-Xin (WTX) promotes ferroptosis via the PI3K/AKT pathway, while SSG (Shenqi Sanye Granules) inhibits CRC proliferation through Hmox1-mediated ferroptosis pathways (98, 99). These studies highlight TCM’s potential to combine direct tumor inhibition with immune modulation and ferroptosis, offering innovative strategies for cancer immunotherapy. Its holistic, multi-target approach, particularly in inducing ferroptosis and modulating immune responses, represents a promising avenue for advancing CRC treatment.

In addition, nanomaterials are increasingly being used in ferroptosis-mediated immunotherapy. Nanotechnology enhances the targeting, controlled release, and stability of drugs while minimizing side effects in CRC treatment (79). Smart nanocarriers, designed to respond to TME-specific signals, enable precise drug delivery and improved therapeutic outcomes. For example, Li Q et al. investigated leukocyte membrane-encapsulated polylactic acid-hydroxyacetic acid-encapsulated glycyrrhizinopropyls (GCMNPs), which, when combined with ferumoxyto, an iron supplement, decreased GPX4 expression, led to increased levels of lipid peroxidation, and induced ferroptosis (100). In contrast, Xin Y et al. designed a fish oil-based microemulsion system, which can effectively orally deliver the inhibitory peptide OPBP-1, which blocks PD-1/PD-L1, to induce ferroptosis in tumor cells and also synergistically interact with ICIs to further enhance the antitumor effect (101). Furthermore, advanced dual-targeting systems, such as GOx@FeNPs, leverage photothermal therapy (PTT) and ferroptosis synergy to boost CRC cell immunogenicity, enhancing dendritic cell (DC) maturation and CD8+ T-cell activation. Combined with αPD-L1, this system showed remarkable efficacy in CRC growth suppression (102). Li et al. further developed a pH- and glutathione-responsive nanodrug delivery system (PMDC NPs) targeting dihydroartemisinin (DHA) and CORM-401, inducing oxidative stress-related apoptosis and ferroptosis while triggering immunogenic cell death (ICD). These nanoparticles activate immune effector cells, including CD4+ and CD8+ T cells, mature DCs, and M1 tumor-associated macrophages (TAMs), while inhibiting Tregs and M2 TAMs (103). Overall, these innovations highlight nanotechnology’s pivotal role in ferroptosis-based CRC immunotherapy, offering potential for overcoming resistance, enhancing efficacy, and enabling personalized treatment strategies. This progress establishes a foundation for future precise and effective CRC therapies. In conclusion, recent research underscores the diversity of colorectal cancer (CRC), highlighting its potential as a natural tumor-suppressing process and a targetable pathway to overcome therapeutic resistance. These advancements offer critical insights into CRC pathogenesis and reveal promising strategies to leverage ferroptosis for enhanced treatment outcomes. Comprehensive understanding—from identifying genetic traits to deploying novel drug interventions—could support the development of precise and effective therapeutic strategies. Such approaches may aid in preventing tumor progression and overcoming CRC resistance. **Table 1** summarizes the application of ferroptosis inducers with ICIs in CRC.

**Table 1:** Application of ferroptosis in the treatment of CRC.

Drugs Name	Target	Mechanism of ferroptosis	Ref.
ACADSB	GPX4	ACADSB induces ferroptosis of CRC cells by negatively regulating GPX4	(104)
Andrographis	β-catenin/Wnt-signaling pathways	Activation of ferroptosis and suppression of β-catenin/Wnt-signaling pathways were the key mediators for the anticancer and chemosensitizing effects of andrographis	(105)
Lipocalin 2	GPX, system Xc^−^	Overexpression of Lipocalin 2 inhibits ferroptosis and promotes CRC progression	(106)
Cisplatin	GPX4	Depletion of reduced glutathione caused by cisplatin and the inactivation of GPX4 plays the vital role in CRC cells to induce ferroptosis	(90)
Cetuximab	NRF2, ROS	Cetuximab inhibits Nrf2/HO-1 pathway to promote ferroptosis in CRC	(107)
Tagitinin C	NRF2/HO-1, lipid peroxidation	Tagitin C activates NRF2/HO-1 pathway to induce ferroptosis	(108)
Sulfasalazine (SSZ)	System Xc^-^GSH	Inhibiting plasma membrane Cys transporter Xc (-) and effectively depleting cellular GSH	(109)
IMCA	SLC7A11	IMCA induces ferroptosis mediates by SLC7A11 through the AMPK/mTOR pathway	(110)
Hydroxy camptothecincinnamaldehyde loaded nanoparticles(PCH)	ROS	Producing ROS	(111)

## Future prospects and conclusions

Ferroptosis as an emerging mechanism of programmed cell death shows promise in CRC therapy (112). It triggers cancer cell death through accumulation of ferroptosis, depletion of GSH and increased lipid peroxidation, and in the process triggers an anti-tumor immune response. In particular, DAMPs released during ferroptosis can activate DCs and CD8+ T cells, thus enhancing immune surveillance of tumors and making it an effective adjunct to immunotherapy. Thus, ferroptosis provides an effective immune adjunct to CRC therapy.

Nevertheless. The physiological roles and regulatory mechanisms of ferroptosis remain many unanswered questions, and in-depth exploration of its regulatory network, dynamic changes in lipid metabolism, and synergistic effects with other cell death modalities, such as apoptosis and autophagy, will reveal its dynamics in the tumour microenvironment (TME), especially in the regulation of lipid metabolism and immune cell activity. It is a promising strategy to develop specific ferroptosis inducers targeting these molecular mechanisms, which should not only have high selectivity and low toxicity, but also good tissue penetration to effectively reach the tumor site. In addition, the optimal combination in the combination therapy strategy deserves further exploration. Existing studies have shown that ferroptosis inducers in combination with ICIs (e.g., PD-1/PD-L1 inhibitors) can activate both innate and adaptive immunity, thereby substantially enhancing the anti-tumor effect. However, the optimal drug combinations, dosages, and timing of administration need to be formulated through in-depth studies for the wide application of such combination therapy regimens in the clinic. Especially in terms of individualized treatment, the tumor microenvironment and immune status of different CRC patients vary greatly, and how to combine these features to design personalized treatment plans will be an important step toward achieving precision therapy. Conducting clinical trials to validate the safety and efficacy of these treatment strategies is essential. In addition to the need to focus on the toxic side effects of ferroptosis inducers, their efficacy in patients with different stages of CRC should be evaluated. Through these multilevel studies, ferroptosis is expected to become an important component of CRC treatment, providing patients with more therapeutic options and significantly improving their prognosis. In this context, traditional Chinese medicine (TCM) demonstrates significant potential in CRC immunotherapy due to its multi-target mechanisms and low side effects. Active compounds like ginsenosides can regulate ferroptosis-related pathways and improve the immune state of the tumor microenvironment (TME). However, TCM faces challenges such as low bioavailability, complex compound structures, and unclear mechanisms. Future research should focus on elucidating TCM molecular mechanisms and optimizing its synergy with immunotherapy and ferroptosis inducers to enhance therapeutic design and overcome existing limitations. Moreover, nanotechnology offers innovative approaches to CRC immunotherapy. Nano-delivery systems improve drug stability and targeting, addressing issues like uneven biodistribution and reducing side effects. Functionalized nanoparticles enable controlled release and TME-specific targeting, enhancing the efficacy of combined therapies. In summary, ferroptosis introduces a novel perspective and strategy for CRC immunotherapy. Future advancements require interdisciplinary collaboration, integrating immunotherapy, ferroptosis inducers, TCM, and nanotechnology to refine treatments and accelerate clinical translation. Multicenter clinical trials are essential to validate efficacy and safety, while personalized strategies based on patient-specific factors can achieve precision treatment and better outcomes. These efforts promise to offer more therapeutic options, significantly improving survival rates and quality of life for CRC patients.
